# Preoperative automated fibre quantification predicts postoperative seizure outcome in temporal lobe epilepsy

**DOI:** 10.1093/brain/aww280

**Published:** 2016-11-15

**Authors:** Simon S Keller, G Russell Glenn, Bernd Weber, Barbara A K Kreilkamp, Jens H Jensen, Joseph A Helpern, Jan Wagner, Gareth J Barker, Mark P Richardson, Leonardo Bonilha

**Affiliations:** 1 1 Department of Molecular and Clinical Pharmacology, Institute of Translational Medicine, University of Liverpool, UK; 2 2 Department of Neuroradiology, The Walton Centre NHS Foundation Trust, Liverpool, UK; 3 3 Department of Basic and Clinical Neuroscience, Institute of Psychiatry, Psychology and Neuroscience, King’s College London, UK; 4 4 Center for Biomedical Imaging, Medical University of South Carolina, Charleston, USA; 5 5 Department of Radiology and Radiological Sciences, Medical University of South Carolina, Charleston, USA; 6 6 Department of Neurosciences, Medical University of South Carolina, Charleston, USA; 7 7 Department of Epileptology, University of Bonn, Germany; 8 8 Department of Neurocognition / Imaging, Life and Brain Research Centre, Bonn, Germany; 9 9 Department of Neurology, Epilepsy Centre Hessen-Marburg, University of Marburg Medical Centre, Germany; 10 10 Department of Neuroimaging, Institute of Psychiatry, Psychology and Neuroscience, King’s College London, UK; 11 11 Engineering and Physical Sciences Research Council Centre for Predictive Modelling in Healthcare, University of Exeter, UK; 12 12 Department of Neurology, Medical University of South Carolina, Charleston, USA

**Keywords:** imaging, outcome, prognosis, seizures, surgery

## Abstract

Approximately one in every two patients with pharmacoresistant temporal lobe epilepsy will not be rendered completely seizure-free after temporal lobe surgery. The reasons for this are unknown and are likely to be multifactorial. Quantitative volumetric magnetic resonance imaging techniques have provided limited insight into the causes of persistent postoperative seizures in patients with temporal lobe epilepsy. The relationship between postoperative outcome and preoperative pathology of white matter tracts, which constitute crucial components of epileptogenic networks, is unknown. We investigated regional tissue characteristics of preoperative temporal lobe white matter tracts known to be important in the generation and propagation of temporal lobe seizures in temporal lobe epilepsy, using diffusion tensor imaging and automated fibre quantification. We studied 43 patients with mesial temporal lobe epilepsy associated with hippocampal sclerosis and 44 healthy controls. Patients underwent preoperative imaging, amygdalohippocampectomy and postoperative assessment using the International League Against Epilepsy seizure outcome scale. From preoperative imaging, the fimbria-fornix, parahippocampal white matter bundle and uncinate fasciculus were reconstructed, and scalar diffusion metrics were calculated along the length of each tract. Altogether, 51.2% of patients were rendered completely seizure-free and 48.8% continued to experience postoperative seizure symptoms. Relative to controls, both patient groups exhibited strong and significant diffusion abnormalities along the length of the uncinate bilaterally, the ipsilateral parahippocampal white matter bundle, and the ipsilateral fimbria-fornix in regions located within the medial temporal lobe. However, only patients with persistent postoperative seizures showed evidence of significant pathology of tract sections located in the ipsilateral dorsal fornix and in the contralateral parahippocampal white matter bundle. Using receiver operating characteristic curves, diffusion characteristics of these regions could classify individual patients according to outcome with 84% sensitivity and 89% specificity. Pathological changes in the dorsal fornix were beyond the margins of resection, and contralateral parahippocampal changes may suggest a bitemporal disorder in some patients. Furthermore, diffusion characteristics of the ipsilateral uncinate could classify patients from controls with a sensitivity of 98%; importantly, by co-registering the preoperative fibre maps to postoperative surgical lacuna maps, we observed that the extent of uncinate resection was significantly greater in patients who were rendered seizure-free, suggesting that a smaller resection of the uncinate may represent insufficient disconnection of an anterior temporal epileptogenic network. These results may have the potential to be developed into imaging prognostic markers of postoperative outcome and provide new insights for why some patients with temporal lobe epilepsy continue to experience postoperative seizures.

## Teaser

Around half of all patients fail to achieve seizure freedom after temporal lobe resection. Keller *et al.* show that postoperative seizures after amygdalohippocampectomy are associated with regional tissue abnormalities and insufficient resection of temporal lobe white matter tracts. These results hold promise as imaging prognostic markers of postoperative seizure outcome.

## Introduction

Epilepsy is the most common serious neurological disorder, affecting over 50 million people worldwide (Ngugi *et al.*, 2010; Neligan *et al.*, 2012). Approximately 30% of all patients with a diagnosis of epilepsy will develop chronic pharmacoresistant epilepsy (Sander and Shorvon, 1996). Temporal lobe epilepsy (TLE) is the most common pharmacoresistant focal epilepsy disorder (Semah *et al.*, 1998; Engel, 2001) and is potentially remediable by neurosurgical intervention.

In the only randomized controlled trial of surgery for refractory TLE, it was reported that surgical intervention is significantly superior for the attainment of seizure freedom 1 year after surgery compared to continuing pharmacological treatment (Wiebe *et al.*, 2001); at 1 year, 58% of patients receiving surgery were free from seizures impairing awareness and 38% were free from any seizure-related symptom, whereas only 8% were seizure-free in the non-surgical control group. There are contrasting reports regarding the proportion of patients attaining seizure freedom after temporal lobe surgery for refractory seizures, which may range from 35 to 80% (Berkovic *et al.*, 1995; Wiebe *et al.*, 2001; McIntosh *et al.*, 2004; de Tisi *et al.*, 2011; Giulioni *et al.*, 2013; Hemb *et al.*, 2013). The most significant contributions to this variance are likely to be time to postoperative follow-up (longer follow-up is associated with lower seizure-free rate) and definition of seizure freedom (complete seizure freedom is associated with lower seizure-free rate relative to freedom from disabling seizures only). The reasons underlying persistent postoperative seizures in patients who are seemingly excellent candidates for temporal lobe surgery are unknown. Although patients with TLE and neuroradiological evidence of hippocampal sclerosis have improved postsurgical outcomes relative to patients with TLE and no MRI lesion (Berkovic *et al.*, 1995; McIntosh *et al.*, 2004), between two-thirds and one-half of patients with hippocampal sclerosis will experience postoperative seizures (Berkovic *et al.*, 1995; Janszky *et al.*, 2005). Current suggestions for why these persistent postoperative seizures occur include a combination of insufficient resection of mesial temporal lobe tissue (Bonilha *et al.*, 2004; Bonilha and Keller, 2015), mesial temporal lobe pathology existing outside the margins of resection (Babb *et al.*, 1984; Holmes *et al.*, 2000; Prasad *et al.*, 2003; Keller *et al.*, 2007), contralateral temporal lobe seizure involvement (Hennessy *et al.*, 2000; Lin *et al.*, 2005; Keller *et al.*, 2007), occult extra-temporal lobe involvement, including temporal-plus epilepsy (Sisodiya *et al.*, 1997; Ryvlin and Kahane, 2005; Kahane *et al.*, 2015; Barba *et al.*, 2016), structural network alterations (Bonilha *et al.*, 2015; Keller *et al.*, 2015*b*), and atypical subtypes of TLE that may be particularly resistant to conventional temporal lobe surgery (Blumcke *et al.*, 2007; Thom *et al.*, 2010; Bonilha *et al.*, 2012). The development of predictive biomarkers for the future success of surgical intervention in epilepsy represents an important research endeavour, particularly as a reliable prognostic marker could inform patient clinical management and surgical decision-making.

As non-invasive imaging techniques improve, there is increasing interest in modelling brain connectivity. This endeavour is providing new insights into the structural and functional organization of the human brain, as well as how alterations in connectivity underlie neurological disorders. Understanding brain connectivity in epilepsy is particularly important given that even focal seizures may be generated in context of distributed epileptogenic brain networks (Richardson, 2012; Bernhardt *et al.*, 2015). Diffusion tensor imaging (DTI) techniques permit the reconstruction of white matter tract bundles, which form the connections between cortical regions within structural networks. There has been increasing application of tractography techniques to study DTI scalar metric alterations for reconstructed white matter tracts in patients with TLE, with a particular focus on tracts within and connecting to the temporal lobe (Bernhardt *et al.*, 2013). However, there is a paucity of data on the relationship between preoperative DTI tractography and postoperative seizure outcome after temporal lobe resection. This may be partly due to the fact that sophisticated DTI acquisitions are not incorporated into routine preoperative evaluation in a clinical setting. However, the application of graph theoretical methods to determine alterations in structural network topology is growing in TLE (Bernhardt *et al.*, 2015), and there have been recent attempts to correlate preoperative structural connectomes with postoperative seizure outcome in small groups of patients with TLE (Bonilha *et al.*, 2013, 2015; Munsell *et al.*, 2015). Despite the interest in developing potential prognostic markers of outcome using preoperative connectomes, the underlying biological significance and anatomical specificity of such data are difficult to interpret.

Automated fibre quantification (AFQ) is a DTI tractography technique that permits a comprehensive analysis of tissue characteristics along the length of white matter tract bundles (Yeatman *et al.*, 2012). This approach offers a potentially more sensitive measure of neuroanatomical white matter alterations in patients with neurological disorders than whole-tract approaches, as it considers regional intratract tissue characteristics. Tissue characteristics may vary considerably along a tract (Johnson *et al.*, 2013), which conventional DTI analyses of whole tract mean diffusion measures are unable to consider. Furthermore, it is likely that at least some pathological alterations in TLE occur in circumscribed regions of tracts and not along entire tracts. Such anatomical specificity could potentially improve the detection of anatomical prognostic markers of treatment outcome in patients with TLE.

In the present study, we applied AFQ to preoperative DTI in patients with TLE who underwent surgical treatment and postoperative follow-up, with a primary goal of identifying preoperative diffusion markers of postoperative seizure outcome. We focused on three temporal lobe tract bundles that are known to be important in the generation and propagation of temporal lobe seizures and susceptible to pathological alterations in refractory TLE: the fimbria-fornix (Concha *et al.*, 2005, 2009, 2010), parahippocampal white matter bundle (McDonald *et al.*, 2008; Yogarajah *et al.*, 2008; Ahmadi *et al.*, 2009; Keller *et al.*, 2012) and uncinate fasciculus (Diehl *et al.*, 2008; Lin *et al.*, 2008; Ahmadi *et al.*, 2009). A secondary goal of the present study was to determine whether extent of resection of the temporal lobe tract bundles was associated with seizure outcome. While there are several studies that have addressed whether the general extent of resection is associated with outcome based on analysis of conventional (e.g. T_1_-weighted) MRI scans (Jack *et al.*, 1988; Kanner *et al.*, 1995; Salanova *et al.*, 1996; Hardy *et al.*, 2003; Bonilha *et al.*, 2004; Joo *et al.*, 2005; Keller *et al.*, 2015*b*), there has to date been no assessment of the relationship between seizure outcome and extent of white matter tract resection.

## Materials and methods

### Participants

From a series of 115 consecutive cases with TLE and hippocampal sclerosis being considered for temporal lobe surgery at University Hospital Bonn, Germany, between 2006 and 2011, 43 patients were studied in this investigation [27 left TLE, 16 right TLE; 23 females, 20 males; mean age 39.7 years, standard deviation (SD) 12.6]. All patients in the wider cohort had a comprehensive presurgical evaluation at University Hospital Bonn, which included clinical assessment of seizure semiology, interictal EEG, long-term video EEG monitoring, if clinically necessary additional invasive electrophysiological investigations, diagnostic MRI [T_1_-weighted, T_2_-weighted and T_2_ fluid attenuated inversion recovery (FLAIR) scans], and neuropsychological assessment (Kral *et al.*, 2002). For each patient, hippocampal sclerosis was identified by an expert neuroradiologist with considerable experience of lesion diagnosis in epilepsy, and was defined by hippocampal volume loss and internal structure disruption on T_1_-weighted scans, and/or hyperintensities on T_2_-weighted and FLAIR images. The 43 selected patients fitted the following inclusion criteria for the present study: (i) availability of high quality preoperative DTI data suitable for deterministic tractography; (ii) no evidence of bilateral hippocampal sclerosis or of a secondary extrahippocampal lesion that may have contributed to seizures; (iii) underwent amygdalohippocampectomy (Bien *et al.*, 2013); (iv) diagnosis of hippocampal sclerosis on histopathological assessment; and (v) standardized postoperative outcome assessment. Histological confirmation of hippocampal sclerosis was performed using the now standardized International League Against Epilepsy (ILAE) classification (Blumcke *et al.*, 2013). Postsurgical seizure outcome was assessed using the ILAE outcome classification system (Wieser *et al.*, 2001). All patients had a minimum of 12 months and a mean of 24 months postoperative follow-up. We additionally studied a series of 44 neurologically healthy controls (28 females, 16 males; mean age 38.0 years, SD 14.0).

### MRI acquisition

All study participants underwent MRI at the Life and Brain Center in Bonn on a 3 T scanner (Magnetom Trio, Siemens). An eight-channel head coil was used for signal reception. T_1_-weighted magnetization-prepared rapid gradient-echo images (160 slices, repetition time = 1300 ms, inversion time = 650 ms, echo time = 3.97 ms, voxel size 1.0 × 1.0 × 1.0 mm, flip angle 10°) were acquired for all patients prior to surgery and all controls. Postoperative T_1_-weighted data were acquired for 33 patients. Diffusion-weighted data (diffusion-weighted single shot spin-echo echo-planar imaging sequence, repetition time = 12 s, echo time = 100 ms, 72 axial slices, voxel size 1.726 × 1.726 × 1.7 mm, no cardiac gating, GRAPPA acceleration factor 2) was also acquired for all patients preoperatively and controls. Diffusion gradients were equally distributed along 60 directions (b-value = 1000 s/mm^2^). Additionally, six datasets with no diffusion weighting (b-value = 0 s/mm^2^) (b0 images) were acquired in an interleaved fashion, with one b0 dataset preceding each block of 10 diffusion-weighted images.

### Image analysis

Automatic segmentation and volume estimation of hippocampal and extrahippocampal subcortical structures was performed using Freesurfer software (Fischl, 2012) applied to the T_1_-weighted images, as previously described (Keller *et al.*, 2012). For DTI analysis, motion correction was performed on the diffusion-weighted data using SPM8 (Wellcome Trust Centre for Neuroimaging, London, UK) using the initial b0 image for each subject as a reference, with subsequent b0 images being co-registered with a 12-parameter affine transformation. The transformation for each b0 image was applied to the 10 subsequent diffusion-weighted images and the diffusion encoding vectors were corrected for all rotations of the image volume (Leemans and Jones, 2009). After co-registration, an average b0 dataset was created, and the full DTI dataset was processed using the AFQ image analysis pipeline (https://github.com/jyeatman/AFQ).

AFQ performed a series of automated steps, including additional motion correction for each of the individual diffusion-weighted images and voxel-wise estimation of the diffusion tensor. Brain masks were created within AFQ using an automated brain extraction tool (Smith, 2002) and tractography was performed within the brain mask using the Euler method with a step size of 1 mm, an angle threshold of 35°, and a minimum tract length of 20 mm (Basser *et al.*, 2000). Following tractography AFQ performed a non-linear normalization of the average b0 dataset for each subject to the International Consortium for Brain Mapping template. This non-linear transformation was then used to map standardized white matter regions of interest from the template to the diffusion images to demarcate common anatomical landmarks in each subject. AFQ then automatically segmented the tractography data into fibre bundles of interest using the template-defined regions of interest as the starting and ending point for each fibre bundle. Once fibre bundles were segmented, AFQ identified the core region of each bundle and calculated along-the-tract diffusion profiles by interpolating a fixed number of sections along the long-axis of each tract. Thus to accommodate intersubject variability in tract distributions, AFQ normalized each subject’s tractography-identified fibre bundles at their endpoints using standardized regions of interest while allowing them to vary in between, such that each interpolated section (for example, start, middle, and end) was considered to be the same and compared between subjects. This is distinctly different from voxel-wise approaches, which assume that each voxel represents the same type or region of tissue after normalization.

Fibre bundles were selected based on their hypothesized roles in TLE, and included the fimbria-fornix, mesial temporal portion of the cingulum (referred to as the ‘cingulum hippocampus’ in context of AFQ software, here after referred to as the parahippocampal white matter bundle), and uncinate fasciculus. For segmentation of the fimbria-fornix, we implemented an in-house algorithm using AFQ’s routine [see Supplementary material and Glenn *et al.* (2016)]. Each fibre bundle was interpolated along 100 sections and along-the-tract profiles were reconstructed for mean diffusivity and fractional anisotropy for both left- and right-sided pathways. For patients with TLE, tract profiles were separated into ipsilateral and contralateral sides, and for controls, tract profiles for left- and right-side pathways were combined. Tract profiles were excluded in instances where AFQ could not reconstruct the white matter pathways (Johnson *et al.*, 2013).

### Statistical analysis of tract profiles

**Figure 1:**
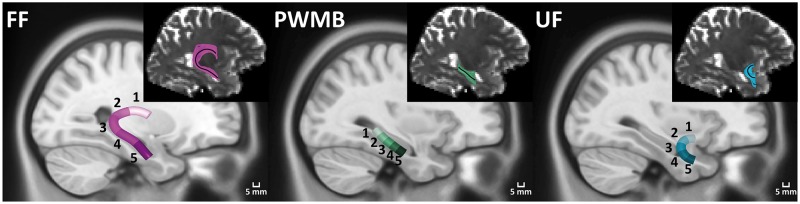
**Anatomical location of fibre bundle regions of interest used for statistical comparison.** The inset for each fibre bundle illustrates representative tracts reconstructed for a single subject, with the solid black line indicating the AFQ-identified tract core used for calculation of the tract profiles. Tract cores for each subject are mapped to a template image and averaged to indicate the group-wise representation of each fibre bundle. For statistical comparison, each fibre bundle is divided into five regions of interest by averaging every 20 consecutive tract sections. Region of interest numbers correspond to the regions of interest used in Fig. 2 and Supplementary Table 1. FF = fimbria-fornix; PWMB = parahippocampal white matter bundle; UF = uncinate fasciculus.

Tract profiles were compared between healthy controls, patients rendered completely seizure free (ILAE 1) and patients with persistent postoperative seizure-related symptoms (ILAE 2–6). For statistical analysis, individual tract profiles were averaged over five regions of interest consisting of sets of 20 consecutive sections. Comparisons were performed with a two sample *t*-test and multiple comparisons were corrected for using the false discovery rate procedure (Benjamini and Hochberg, 1995). Effect size was quantified using Cohen’s *d* parameter. The regions of interest used are illustrated in Fig. 1 along with representative tract profiles from a single patient with TLE. To illustrate the anatomical location of the observed differences, a section-wise *t-*score plot was reconstructed.

### Development of potential biomarker assays

To test the potential clinical applicability of the preoperative diffusion-weighted data, receiver operating characteristic (ROC) curves for the along-the-tract profiles were calculated. For the ROC curves, regions of interest were selected along each pathway based on observed differences in tissue characteristics, and individual tract profiles were averaged over each region of interest. Sensitivity and specificity were assessed for group-wise separations between TLE and control groups as well as between patient outcome groups for incrementally decreasing values of the test parameter. The regions of interest used to distinguish between patient outcome groups were also pooled to test the combination of multiple classifiers for outcome prediction.

### White matter bundle resection analysis

Of the 43 patients studied, 33 received postoperative structural imaging. Lacunar maps of the resected tissue volumes were traced on postoperative T_1_-weighted images as previously described (Keller *et al.*, 2015*b*), and postoperative images were normalized to the template used by AFQ using the Clinical Toolbox for SPM (Rorden *et al.*, 2012) (https://www.nitrc.org/projects/clinicaltbx/) with enantiomorphic normalization to account for loss of the resected tissue (Nachev *et al.*, 2008). Individual fibre bundles were then mapped to the template using the AFQ-identified non-linear deformation, and tract profiles were reconstructed using AFQ’s routine over the normalized, binary lacunar maps. Thus, tract profiles were created by calculating the proportion of the resected fibre bundle at a given section overlapping with the resected tissue. The total proportion of an individual fibre bundle resected was then calculated by averaging over all sections. Comparisons of fibre bundle resections between patient outcome groups were then made with a two sample *t*-test, correcting for multiple comparisons using the false discovery rate correction. Fibre bundle resection maps were created using a two-step procedure. First, individual bundle resection maps were created by intersecting the binary mask of the reconstructed fibre bundles with the normalized lacunar maps of the resected tissue for each patient. Subsequently the individual bundle resection maps were averaged, taking into account ipsilateral and contralateral distinctions by flipping the ipsilateral side to the left hemisphere. For anatomical reference, fibre bundle distribution maps were calculated for the control group by averaging the binary masks of the left-sided fibre bundles.

## Results

### Clinical information

Of the 43 patients included in this study, 22 (51.2%) patients had an excellent postoperative seizure outcome (ILAE 1) and 21 (48.8%) had a suboptimal outcome (ILAE 2–5). No patient experienced worsening seizures after surgery (ILAE 6). A breakdown of clinical variables according to outcome groups is provided in Table 1. There were no significant differences between outcome groups with respect to patient age, age of onset of epilepsy, duration of epilepsy, seizure frequency, a history of childhood febrile seizures, or ILAE classification of hippocampal sclerosis. There were a greater proportion of males who were rendered seizure free relative to females (*P = *0.03).

**Table 1:** Clinical information with respect to outcome

	ILAE 1	ILAE 2+	Significance
*n*	22 (51.2%)	21 (48.8%)	-
Outcomes	1 = 22	2 = 5 3 = 7 4 = 8 5 = 1 6 = 0	-
ILAE histopathology	ILAE I = 20 ILAE 2 = 2 ILAE 3 = 0	ILAE I = 17 ILAE 2 = 4 ILAE 3 = 0	χ^2 ^= 0.9, *P = *0.35
Invasive recordings, no/yes	16/6	14/7	χ^2 ^= 0.2, *P = *0.67
Left/right TLE	11/11	16/5	χ^2 ^= 3.2, *P = *0.12
Female/male	8/14	15/6	χ^2 ^= 5.3, *P = *0.03
Febrile seizures, no/yes	15/7	14/7	χ^2 ^= 0.01, *P = *0.59
Age	38.8 (11.3)	40.6 (13.9)	F = 0.22, *P = *0.64
Onset	16.05 (11.49)	15.6 (10.5)	F = 0.02, *P = *0.89
Duration	22.7 (13.9)	25.0 (15.8)	F = 0.25, *P = *0.62
Seizure frequency	8.8 (18.7)	4.2 (2.3)	F = 1.27, *P = *0.27

Outcome, side of TLE, sex, and incidence of febrile seizures are number. Age, age of onset of epilepsy, preoperative duration of epilepsy, and preoperative seizure frequency are median (and interquartile range).

### Volumetric comparisons

Table 2 provides information on hippocampal, whole grey matter and whole white matter volume comparisons between patients and controls, and between patient outcome groups. Hippocampal volumes were significantly smaller ipsilateral to the side of intended resection relative to healthy controls. There was no evidence of bilateral hippocampal atrophy in patients relative to controls. Whole grey and white matter volumes were not significantly different between patients and controls. Furthermore, there were no differences in ipsilateral or contralateral hippocampal, grey matter, or white volumes between patients with an excellent or suboptimal outcome. There were also no significant differences in extrahippocampal subcortical volumes between outcome groups (Supplementary material).

**Table 2:** Comparison of hippocampal, whole grey matter and whole white matter volumes between groups

	Controls	Left TLE	Right TLE	Significance
Left hippocampal volume	*3840 (382)	*^,#^3085 (783)	^#^3619 (388)	F = 16.48: *^,#^*P* < 0.001
Right hippocampal volume	*3831 (380)	^#^3762 (574)	*^,#^3091 (548)	F = 14.64: *^,#^*P* < 0.001
Whole grey matter volume	567 817 (63 127)	522 483 (96 859)	538 986 (80 643)	F = 2.96: *P > *0.05
Whole white matter volume	586 782 (56 452)	549 447 (80 701)	560 390 (58 475)	F = 2.96: *P > *0.05
	-	**ILAE 1**	**ILAE 2+**	
Ipsilateral hippocampal volume	-	3329 (729.7)	3120 (499.0)	F = 0.96 *P = *0.41
Contralateral hippocampal volume	-	4289 (703)	4156 (603)	F = 0.44 *P = *0.51
Whole grey matter volume	-	462 204 (74 066)	449 097 (80 296)	F = 0.31 *P = *0.58
Whole white matter volume	-	474 268 (72 807)	476 185 (79 811)	F = 0.01 *P = *0.94

*Top*: Comparisons between controls and patients with unilateral TLE. Asterisks and hash symbols indicated corresponding comparisons.

*Bottom*: Comparisons between patients with an excellent postoperative outcome (ILAE 1) and suboptimal outcome (ILAE 2+). Values are mean volume (mm^3^) (and SD).

F = main ANOVA value; *P* = significance level of corresponding comparison; sig = significance.

### Automated fibre quantification comparisons

The parahippocampal white matter bundle was identified bilaterally in all subjects. The uncinate fasciculus was identified bilaterally in all controls and the side ipsilateral to seizure onset in all patients with TLE. On the contralateral side, the uncinate fasciculus was identified in 21 of 22 (95%) patients in the ILAE 1 group and 20 of 21 (95%) patients in the ILAE 2+ group. The fimbria-fornix was identified in 33 of 44 (75%) controls on the left side and 38 of 44 (86%) controls on the right side with no detection bilaterally in four (9%). For the ILAE 1 group, the fimbria-fornix was identified in 19 of 22 (86%) patients on the ipsilateral side and 19 of 22 (86%) on the contralateral side with no detection bilaterally in two (9%). For the ILAE 2+ group, the fimbria-fornix was identified in 19 of 22 (90%) patients on the ipsilateral side and 19 of 22 (90%) on the contralateral side with no detection bilaterally in one (5%).

**Figure 2:**
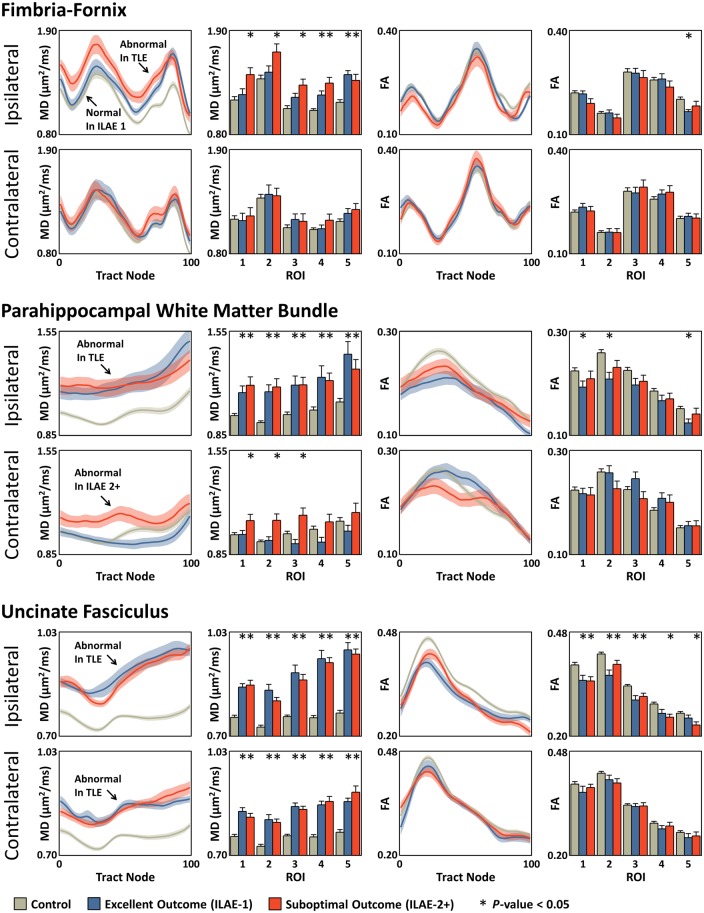
**Mean diffusivity (MD) and fractional anisotropy (FA) tract profiles for mean (± SEM) ipsilateral and contralateral tracts in the ILAE 1 and ILAE 2+ groups relative to controls.** The histograms indicate the average tract profile over a given region of interest. In all cases, increasing tract section corresponds to increasing region of interest number and the regions of interest correspond to those given in Fig. 1. *P-value < 0.05 compared to controls after correcting for multiple comparisons with the FDR procedure. Arrows highlight statistically significantly different regions in the mean diffusivity tract profiles.

**Figure 3:**
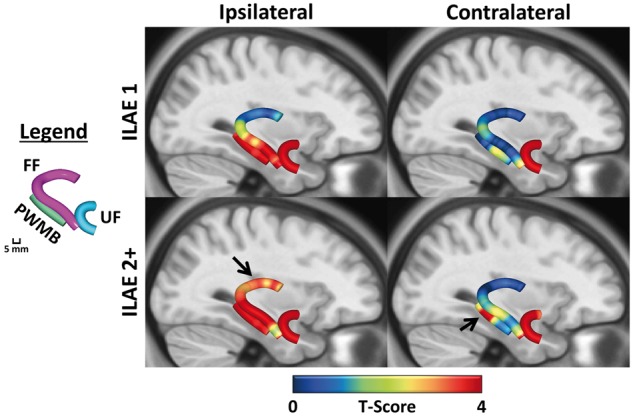
**Section-wise *t*-scores for mean diffusivity tract profiles.** Differences between patient groups and controls are shown projected onto an anatomical template to illustrate the localization of alterations in Fig. 2. Red areas represent significantly increased mean diffusivity in respective patient groups relative to controls. Arrows indicate regions significantly different only in patients with a suboptimal outcome. FF = fimbria-fornix; PWMB = parahippocampal white matter bundle; UF = uncinate fasciculus.

Ipsilateral and contralateral tract profiles for ILAE 1 and ILAE 2+ groups relative to controls are shown in Fig. 2, including corresponding histograms for average tract profiles over each region of interest. Mean diffusivity tract characteristics were generally more revealing than fractional anisotropy characteristics. Mean diffusivity tract profiles were significantly higher in both outcome groups relative to controls along the entire length of the ipsilateral parahippocampal white matter bundle (Fig. 2, middle left) and the uncinate fasciculus bilaterally (Fig. 2, bottom left). Mean diffusivity was also significantly higher for both outcome groups in the ipsilateral fimbria-fornix in regions of interest 4 and 5. Conversely, only ILAE 2+ patients showed evidence of significantly increased mean diffusivity within ipsilateral fornical regions of interest 1–3 (Fig. 2, top left). Controls and ILAE 1 patients had roughly equal mean diffusivity characteristics within these regions of interest. Fornical regions of interest 4 and 5 were located in the mesial temporal lobe, regions of interest 1 and 2 outside the temporal lobe, and region of interest 3 in a transitional region between the two (Fig. 1). Diffusion parameters of the contralateral fimbria-fornix were not altered in patient outcome groups relative to controls. There were additionally significant mean diffusivity alterations only in ILAE 2+ patients located in contralateral parahippocampal white matter bundle regions of interest 1–3 (Fig. 2). To illustrate the location of the observed mean diffusivity differences, section-wise *t*-score plots are reconstructed in Fig. 3. Areas in red represent significant regional increases in mean diffusivity in the respective patient group relative to controls. Arrows indicate the areas exclusively altered only in patients with a suboptimal seizure outcome.

No significant alterations in contralateral fractional anisotropy tract characteristics were observed in patient groups relative to controls. Both patient outcome groups had reduced fractional anisotropy of the ipsilateral uncinate fasciculus through the length of the tract, but only significantly so in regions of interest 4 and 5 (increasingly anterior temporal) for ILAE 2+ patients (Fig. 2, bottom right). The increase in mean diffusivity exclusively in ILAE 2+ patients in the ipsilateral dorsal fornix and contralateral parahippocampal white matter bundle were mirrored by a non-significant reduction in fractional anisotropy in the same regions (Fig. 2). Effect sizes for fractional anisotropy were generally smaller than the corresponding changes in mean diffusivity. The results from Fig. 2 are tabulated in the Supplementary material.

### Receiver operating characteristic curves and outcome prediction

**Figure 4:**
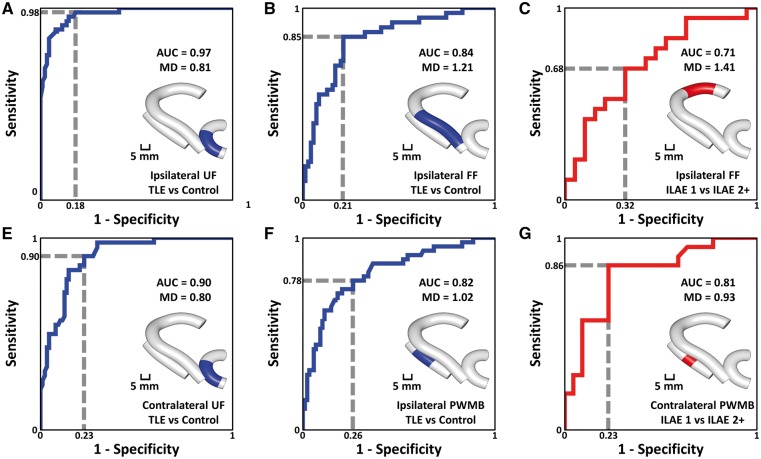
**ROC curves.** In all cases, blue indicates separation between patient and control groups and red indicates separation between patient outcome groups. The area under curve (AUC) is used to assess quality of the ROC curves and the dashed line gives example sensitivity and 1-specificity calculations. MD represents mean diffusivity and the value indicates the corresponding test threshold in units of (µm^2^/ms). The inset for each curve indicates the location of the region of interest used to calculate the ROC curve, which was selected based on observed group differences in mean diffusivity. FF = fimbria-fornix; PWMB = parahippocampal white matter bundle; UF = uncinate fasciculus.

**Figure 5:**
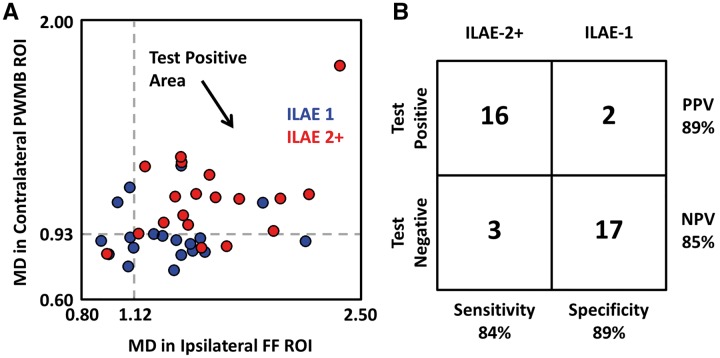
**Combining ipsilateral dorsal fimbria-fornix and contralateral parahippocampal white matter bundle mean diffusivity values increases the sensitivity and specificity for separating patient outcome groups.** (**A**) Mean diffusivity (MD) values in the ipsilateral dorsal fornix and contralateral parahippocampal white matter bundle (PWMB) are plotted on the *x*- and *y*-axes, respectively, for all patients in the ILAE 1 group (blue) and ILAE 2 group (red) using the regions of interest indicated for the respective tracts in Fig. 4C and G. A combined test was used to separate groups for patients with mean diffusivity (MD) >1.12 µm^2^/ms in the ipsilateral fornix and mean diffusivity >0.93 µm^2^/ms in the contralateral parahippocampal white matter bundle indicated by the grey dashed lines with positive test values occurring in the upper right-hand quadrant (black arrow). (**B**) Sensitivity, specificity, positive predictive value (PPV), and negative predictive value (NPV) indicate test performance, illustrating the potential clinical applicability for surgical outcome prediction. FF = fimbria-fornix; PWMB = parahippocampal white matter bundle; ROI = region of interest; UF = uncinate fasciculus.

ROC curves for selected regions of interest are shown in Fig. 4. The ipsilateral and contralateral uncinate (Fig. 4A and E) demonstrated separation between patient and control groups with area under the curve values of 0.97 and 0.90, respectively. The ipsilateral fimbria-fornix and parahippocampal white matter bundle (Fig. 4B and F) demonstrated separation between patient and control groups with area under the curve values of 0.84 and 0.82, respectively. The contralateral parahippocampal white matter bundle also demonstrated separation between patient outcome groups with an area under the curve value of 0.81 (Fig. 4G), and the ipsilateral fimbria-fornix demonstrated separation between outcome groups with an area under the curve value of 0.71 (Fig. 4C). Sensitivity and specificity were both increased when combining mean diffusivity data from the ipsilateral fimbria-fornix and contralateral parahippocampal white matter bundle for the separation of outcome groups (Fig. 5).

### Extent of tract resection

**Figure 6:**
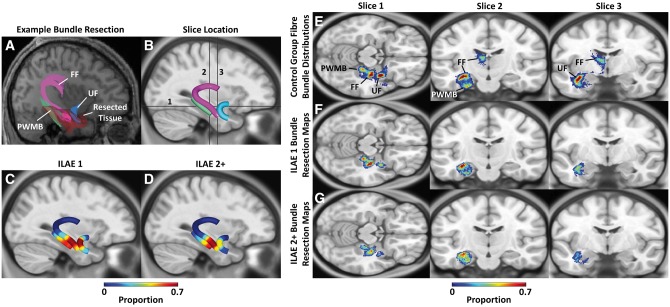
**Fibre bundle resection analysis.** (**A**) Representative tractography data and resection volume overlaid on an individual patient’s T_1_-weighted image illustrate the fibre bundles of interest overlapping with the resected tissue volume in circumscribed regions along each tract. (**B**) Group-wise representation of fibre pathways of interest overlaid on a template image. (**C** and **D**) Section-wise representation of the extent of resected fibre bundles for the ILAE 1 and ILAE 2+ groups, respectively, indicate the region of these tracts typically resected. (**E**) Representative slices for the fibre bundle distributions of the reconstructed tracts in the control group illustrate the anatomical location of the fibre bundles of interest. (**F** and **G**) Fibre bundle resection maps for the ILAE 1 and ILAE 2+ groups, respectively illustrate the proportion of the fibre bundles resected. The location of the representative transverse and coronal slices are given by the black bars in **B**. FF = fimbria-fornix; PWMB = parahippocampal white matter bundle; UF = uncinate fasciculus.

Of the 33 patients with postoperative structural imaging, 17 (51.5%) patients were rendered seizure-free (ILAE 1) while 16 (48.5%) patients experienced persistent postoperative symptoms. Resection maps are shown in Fig. 6. Exemplary tractography and resection data are shown in Fig. 6A, which illustrates the intersections between fibre bundles and resected tissue volume. Section-wise resection maps for the ILAE 1 and ILAE 2+ groups are shown in Fig. 6C and D, respectively. These maps indicate a high probability of anterior fimbria-fornix and parahippocampal white matter bundle resection, and low probability of posterior fimbria-fornix and parahippocampal white matter bundle resection, across all patients. However, outcome group ILAE 1 had high probability of uncinate fasciculus resection, whereas group ILAE 2+ had a lower probability of uncinate resection. Representative transverse and coronal image slices of the left sided fibre bundle distributions for the control group are given in Fig. 6E, demonstrating the anatomical location of the reconstructed fibre bundles. In Fig. 6F and G, voxel-wise resection maps for the reconstructed fibre bundles are indicated for ILAE 1 and ILAE 2+ groups. The location of the image slices are indicated by the black bars in Fig. 6B.

The ILAE 1 group had non-significant increases in the extent of resected fornix-fimbria and parahippocampal white matter bundle relative to the ILAE 2+ group (fimbria-fornix: 20.8 ± 12.6%, 18.3 ± 8.9%; *P = *0.54; parahippocampal white matter bundle: 44.8 ± 27.2%, 33.2 ± 16.8%; *P = *0.23). However, there was a significantly increased proportion of uncinate fasciculus resection in the ILAE 1 group relative to the ILAE 2+ group (41.7 ± 20.9%, 19.7 ± 23.1%; *P = *0.02). For individual uncinate resections, 1 of 17 patients in the ILAE 1 group had proportions of resection less than 0.15 and 9 of 16 patients in the ILAE 2 group had proportions of resection less than 0.15 giving sensitivity and specificity of 56% and 94%, respectively, for identifying the ILAE 2 group based on proportion of uncinate resection.

## Discussion

The primary objective of the present study was to determine preoperative imaging correlates of postoperative seizure outcome in patients with refractory TLE using a novel DTI technique sensitive to the regional tissue characteristics of temporal lobe white matter tract bundles. We report that whilst all patients with TLE show evidence of diffusion abnormalities of the ipsilateral fimbria-fornix, parahippocampal white matter bundle and uncinate fasciculus, only patients with persistent postoperative seizures have circumscribed alterations in two principal regions that are not observed in patients with an excellent postoperative outcome: the dorsal segment of the ipsilateral fornix and the contralateral parahippocampal white matter bundle. Furthermore, we observed that whilst mean diffusivity of the uncinate fasciculus was considerably affected in both patient outcome groups—and could be used to reliably classify patients from controls using ROC curves—the extent of resection of this tract bundle was also significantly related to postoperative outcome. We separate discussion of these findings according to the three tract bundles investigated, before highlighting pertinent methodological issues.

### Fimbria–fornix

DTI studies of patients with TLE frequently reveal diffusion abnormalities of the fornix, particularly in patients with hippocampal sclerosis (Concha *et al.*, 2005, 2009, 2010). In a novel imaging-histological correlational study, it was reported that preoperative diffusion abnormalities of the fimbria-fornix is significantly related to increased extra-axonal fraction, and reduced cumulative axonal membrane circumference and myelin area of the surgically resected tissue (Concha *et al.*, 2010), thus indicating that *in vivo* diffusion alterations in TLE have a histopathological basis. Myelin pathology has also been implicated in fimbria-fornix DTI alterations in animal models of TLE (van Eijsden *et al.*, 2011). In animal studies, excision of the fornix causing denervation of the hippocampus from subcortical (principally thalamic) targets results in hippocampal seizure activity (Buzsaki *et al.*, 1989), a concomitant loss of hippocampal neurons (Lahtinen *et al.*, 1993*b*) and increased hippocampal *N*-methyl-d-aspartate receptor density (Lahtinen *et al.*, 1993*a*), which may reflect a pathological regenerative process that supports the development of limbic epileptogenicity. There is consequently an accumulation of human and animal data providing support for the hypothesis that the fimbria-fornix has an important role in temporal lobe seizures.

Our data indicate that the fimbria-fornix is equally pathological in mesial temporal lobe regions typically resected in patients who later experience postoperative seizure freedom and those with persistent postoperative seizures. However, only patients who continue to experience persistent postoperative seizures show clear circumscribed diffusion abnormalities in fornical regions outside the margins of resection, principally in dorsal regions proximal to the thalamus. This builds significantly on previous pilot work that indicated that patients with TLE and persistent postoperative seizures had reduced grey matter density outside the margins of resection compared to patients who were rendered seizure-free in a group of patients with left TLE who underwent different surgical interventions (Keller *et al.*, 2007). Furthermore, it was recently reported that a suboptimal postoperative seizure outcome was related to altered tissue diffusion characteristics of probabilistic hippocampothalamic pathways, which included the posterior fornical route amongst other anatomical pathways (Keller *et al.*, 2015*b*). Probabilistic seed-target tractography, like the approach employed by Keller *et al.* (2015*b*), is unable to dissect the specific anatomical pathways within structural networks and the specific regions of tracts that may underlie persistent postoperative seizures. Importantly, only by mapping individual tract pathology along the length of each tract, including that of the fornix, were we able to generate predictive markers of outcome. The fimbria-fornix is the principal connector between the posterior mesial temporal lobe and thalamus (Aggleton *et al.*, 1986) and mediates resting-state functional connectivity between the hippocampus and thalamus (Kehoe *et al.*, 2015). It is possible that a more extensive involvement of the fimbria-fornix may reflect a more extensive epileptogenic network, and surgery may not sufficiently disrupt this network in those with persistent postoperative seizures. While our findings may suggest that a more complete posterior resection of the mesial temporal lobe may offer an improved outcome, we do not yet advocate a change in surgical practice based on our preoperative imaging findings. Translation to the clinic would ideally require a clinical trial to investigate whether this approach adds value to the evaluation and outcome of patients being considered for temporal lobe surgery.

### Parahippocampal white matter bundle

The parahippocampal gyrus, particularly the anterior entorhinal and perirhinal regions, plays an important role in the generation and propagation of temporal lobe seizures (Bernasconi *et al.*, 2000; Wennberg *et al.*, 2002; Bartolomei *et al.*, 2005; Benini *et al.*, 2011). Parahippocampal diffusion alterations have been reported in patients with TLE using DTI techniques (McDonald *et al.*, 2008; Yogarajah *et al.*, 2008; Ahmadi *et al.*, 2009; Keller *et al.*, 2012). In the present study, we report that tissue characteristics of the ipsilateral parahippocampal white matter bundle are similarly affected in patients with excellent and suboptimal postoperative outcomes, but diffusion alterations of a circumscribed region of the contralateral parahippocampal white matter bundle was only identified in patients with persistent seizures. This may be a reflection of a bitemporal seizure disorder in some patients with persistent postoperative seizures. Other imaging studies have suggested contralateral mesial temporal alterations in patients with persistent postoperative seizures (Keller *et al.*, 2007, 2015*a*, *b*; Lin *et al.*, 2005), although parahippocampal involvement was not specified, and none of the aforementioned studies have reported predictive value of contralateral mesial temporal alterations for postoperative outcome in individual patients. Detailed electrophysiological investigations of postoperative seizures in patients with TLE and hippocampal sclerosis suggested that 25% of patients have seizure onset in the contralateral temporal lobe (Hennessy *et al.*, 2000). When contralateral parahippocampal white matter bundle and ipsilateral dorsal fornical mean diffusivity measures were combined, we were able to classify postoperative outcome groups with 84% sensitivity and 89% specificity. A bihemispheric mesial temporal-subcortical epileptogenic network may therefore have significance for persistent postoperative seizures in patients with TLE.

### Uncinate fasciculus

We did not find any preoperative uncinate differences between outcome groups; the ipsilateral and contralateral uncinate fasciculi were affected equally across groups, and throughout the length of the uncinate. A previous study has reported mean diffusivity alterations throughout the entire length of the uncinate in patients with TLE (Concha *et al.*, 2012). Other studies also report diffusion alterations of the uncinate in patients with TLE (Diehl *et al.*, 2008; Lin *et al.*, 2008; Ahmadi *et al.*, 2009). The uncinate fasciculus plays an important role in seizure propagation from the temporal lobe to the frontal lobe in patients with TLE as evidenced in electrophysiological studies (Lieb *et al.*, 1991; Mayanagi *et al.*, 1996), and reflected in studies showing interictal hypometabolism in insular-frontal-opercular regions (Engel *et al.*, 1990; Henry *et al.*, 1993; Chassoux *et al.*, 2004). We did, however, identify that patients who were rendered seizure-free had significantly larger resections of the uncinate relative to those with persistent postoperative seizures. This is a new finding that is compatible with the idea of improved disconnection of anterior epileptogenic networks in patients with TLE and an excellent outcome. It has been suggested that anterior temporal lobe regions are epileptogenic in patients with mesial TLE, and resection of the anterior temporal lobe is associated with an improved outcome (Chabardes *et al.*, 2005). However, whether anterior temporal lobectomy provides consistently improved postoperative seizure outcomes relative to amygdalohippocampectomy is an unresolved issue. A review of the literature has indicated that the extent of resection does not necessarily lead to improved postoperative seizure outcome; that patients with significant hippocampal and amygdaloid remnants may experience excellent postoperative seizure outcomes, and that amygdalohippocampectomy and anterior temporal lobectomy do not differ in rates of seizure freedom (Schramm, 2008). We have recently reported that the general extent of resection of mesial temporal lobe tissue—or resection volume of individual mesial temporal structures—did not significantly relate to postoperative outcome in our group of patients (Keller *et al.*, 2015*b*). In the present study, we have provided important new information indicating that what the resection encompasses is more important than the overall extent of resection, with resection of the uncinate fasciculus in particular being an important factor.

### Methodological issues

There are important methodological issues with the present study that warrant discussion.

#### Image analysis

Our preoperative imaging markers of outcome were obtained in analysis of mean diffusivity, with similar non-significant trends in analysis of fractional anisotropy. In a review of DTI studies in TLE, Bernhardt *et al.* (2013) stated that ‘…the effect size of mean diffusivity alterations in TLE seems to decrease as a function of anatomical distance to the temporal lobe, suggesting co-localization of these changes with the seizure focus’. This is entirely consistent with our data. In an early DTI application in TLE, it was shown that mean diffusivity changes occur proximal to the localization of epileptiform EEG abnormalities (Rugg-Gunn *et al.*, 2001). In studies of the epileptogenic hippocampus in TLE, mean diffusivity has been shown to be a more sensitive marker of pathology compared to fractional anisotropy (Assaf *et al.*, 2003; Salmenpera *et al.*, 2006). Temporal lobe mean diffusivity has been shown to be a stronger predictor for the lateralization of the epileptogenic temporal lobe relative to temporal lobe fractional anisotropy (Khan *et al.*, 2014). Despite that whole-brain mean diffusivity and fractional anisotropy may have lateralizing value, mean diffusivity alterations are more restricted to the hippocampus, fornix and cingulum, i.e. limbic pathways (Chiang *et al.*, 2016). The thalamus, which is known to have important roles in seizure initiation in TLE (Keller *et al.*, 2015*b*), has also been reported to have abnormal mean diffusivity but not fractional anisotropy values in some studies (Kim *et al.*, 2010). In a meta-analysis of DTI studies in TLE, it was reported that ipsilateral mean diffusivity alterations show a significantly larger increase in the white matter passing through the temporal lobe than in remote white matter in patients with TLE (Otte *et al.*, 2012). There are certainly significant fractional anisotropy alterations throughout the brain in patients with refractory TLE, both within the temporal lobe and equally beyond the seizure focus (Gross *et al.*, 2006; Bernhardt *et al.*, 2013). However, measures of mean diffusivity appear to be more specific to potentially epileptogenic tissue.

Partial volume effects and restricted tract reconstructions are inherent issues associated with all kinds of tractography approaches, including AFQ. However, AFQ is a fully automated technique that standardizes tracts across subjects, permitting assessment along the length of each tract, which allows for convenient automated group-comparison studies. Lower tract identification rates in the fimbria-fornix may be attributable to the curvature of the tract or contributions of multiple fibre bundle orientations in complex neural tissue (Johnson *et al.*, 2013). These limitations can potentially be overcome with improved image quality (Johnson *et al.*, 2013) or higher order diffusion techniques (Glenn *et al.*, 2016), which can both augment the performance AFQ. Despite the failed reconstruction of fimbria-fornix bundles in a minority of subjects causing a small reduction in our sample size for analysis, we have demonstrated highly significant differences between outcome groups in this region corrected for multiple comparisons in group comparison studies, and as a potential outcome classifier using ROC curves. Of additional note, we had previously performed probabilistic tractography in 46 patients with TLE and hippocampal sclerosis (Keller *et al.*, 2015*b*), whereas in the present study we investigated 43 patients. This is because along-the-fibre quantification, as used in the present study, is new deterministic tractography methodology, and we therefore included only subjects with little to no image artefacts with the goal of minimizing fibre tracking errors. The probabilistic tractography methods used in our previous study have been more systematically tested and are known to be more robust in overcoming minor artefacts. Probabilistic tractography, however, does not permit along-the-fibre quantification, and it is the latter technique as employed in the present study that has identified predictive imaging markers of outcome.

#### Clinical considerations

Although our sample is one of the largest to date that has investigated the relationship between preoperative DTI and postoperative seizure outcome (Bonilha *et al.*, 2013, 2015; Ji *et al.*, 2015; Keller *et al.*, 2015*b*; Munsell *et al.*, 2015), it is small in context of epidemiological studies of outcome, and therefore caution should be exercised when interpreting the relationship between clinical data and outcomes. We do report a significant effect of sex on outcome, with males being more likely to attain complete seizure freedom compared to females, which is consistent with other larger epidemiological studies (Burneo *et al.*, 2006; Aull-Watschinger *et al.*, 2008). A restricted sample size also affects the generalizability of our results with respect to whether presurgical diffusion abnormalities are sufficient to predict outcome or whether outcomes would be improved by adjusting the surgical margins to include a significant proportion of the uncinate fasciculus. We have demonstrated the sensitivity of AFQ in detecting individual diffusion abnormalities and the potential relevance of these specific structural alterations, which may represent a significant step forward in the clinical translation of advanced neuroimaging techniques for predicting surgical outcomes in TLE. However, given that our ROC analyses are based on an arbitrary cut-off level guided by our group comparison findings, and that this is a retrospective study and has the inherent risk of ascertainment bias, it is important to note that these new findings do not currently represent a clinically useful test. An important future step will be to perform a pragmatic prospective study of consecutive patients with consideration of these new findings. Our reasoning for using a fully automated approach is that this method will potentially lend itself to more clinically useful tests in the future. Finally, because of the limited sample size, it was necessary to side flip imaging data to increase outcome group sample size. Therefore, we were unable to investigate whether the side of seizure onset was related to tract characteristics and outcome.

## Conclusion

The reasons underlying persistent postoperative seizures in patients with refractory TLE may be multifactorial and vary between patients. In the present study, we have identified three important factors that contribute to persistent postoperative seizures: (i) diffusion abnormalities of the ipsilateral dorsal fornix outside the future margins of resection; (ii) diffusion abnormalities of the contralateral parahippocampal white matter bundle; and (iii) insufficient resection of the uncinate fasciculus. These results may have the potential to be developed into imaging prognostic markers of postoperative outcome and provide new insights for why some patients with TLE continue to experience postoperative seizures.

## Funding

This work was supported by a UK Medical Research Council grant awarded to S.S.K. (Grant Number MR/K023152/1). G.R.G. is supported by the National Institutes of Health research (Grant Number T32GM008716 to P. Halushka) and the Litwin Foundation. M.P.R. is supported by a UK Medical Research Council Programme Grant (Grant Number MR/K013998/1) and a UK Engineering and Physical Sciences Centre Grant (Grant Number EP/N014391/). M.P.R. and G.J.B. are supported by the National Institute for Health Research (NIHR) Biomedical Research Centre at the South London and Maudsey NHS Foundation Trust.

## Supplementary material

Supplementary material is available at *Brain* online.

## Supplementary Material

Supplementary Data

## Abbreviations

AFQ: automated fibre quantification
DTI: diffusion tensor imaging
ILAE: International League Against Epilepsy
ROC: receiver operating characteristic
TLE: temporal lobe epilepsy
