# Supplementary material for: Preoperative automated fibre quantification predicts postoperative seizure outcome in temporal lobe epilepsy
Source: Brain. 2016 Nov 15;140(1):68–82. doi: 10.1093/brain/aww280 (PMC5226062; doi:10.1093/brain/aww280)
Supplement: Supplementary Data [file aww280_supp.zip › brain-2016-00271-File009.pdf]

Supplementary Table 1: Comparisons of preoperative extrahippocampal volumes between patients with excellent and suboptimal postoperative outcomes.

Structure		Sum of Squares	df	Mean Square	F	Sig.
**ipsi_Lateral_Ventricle**	Between Groups	69844668.73	1	69844668.73	1.574	0.217
	Within Groups	1819025409	41	44366473.38		
	Total	1888870077	42			
**ipsi_Cerebellum_WhiteMatter**	Between Groups	985175.128	1	985175.128	0.228	0.635
	Within Groups	176882727.7	41	4314212.87		
	Total	177867902.8	42			
**ipsi_Cerebellum_Cortex**	Between Groups	5723.438	1	5723.438	0	0.993
	Within Groups	3269497175	41	79743833.53		
	Total	3269502898	42			
**ipsi_Thalamus**	Between Groups	21641.866	1	21641.866	0.013	0.908
	Within Groups	65924580.74	41	1607916.603		
	Total	65946222.61	42			
**ipsi_Caudate**	Between Groups	538518.264	1	538518.264	1.453	0.235
	Within Groups	15194742.85	41	370603.484		
	Total	15733261.12	42			
**ipsi_Putamen**	Between Groups	253569.972	1	253569.972	0.407	0.527
	Within Groups	25561072.03	41	623440.781		
	Total	25814642	42			
**ipsi_Pallidum**	Between Groups	19762.004	1	19762.004	0.217	0.644
	Within Groups	3735019.159	41	91098.028		
	Total	3754781.163	42			

**ipsi_Amygdala**	Between Groups	48863.798	1	48863.798	0.394	0.534
	Within Groups	5090754.202	41	124164.737		
	Total	5139618	42			
**ipsi_Accumbens_area**	Between Groups	3953.42	1	3953.42	0.535	0.469
	Within Groups	303080.254	41	7392.201		
	Total	307033.674	42			
**ipsi_VentralDC**	Between Groups	73.287	1	73.287	0	0.988
	Within Groups	12774504.71	41	311573.286		
	Total	12774578	42			
**contra_Lateral_Ventricle**	Between Groups	46795707.4	1	46795707.4	1.051	0.311
	Within Groups	1825619932	41	44527315.41		
	Total	1872415639	42			
**contra_Cerebellum_WhiteMatter**	Between Groups	462292.246	1	462292.246	0.088	0.768
	Within Groups	214312488.4	41	5227133.862		
	Total	214774780.6	42			
**contra_Cerebellum_Cortex**	Between Groups	6412932.692	1	6412932.692	0.074	0.787
	Within Groups	3554548838	41	86696313.12		
	Total	3560961771	42			
**contra_Thalamus**	Between Groups	6398.509	1	6398.509	0.004	0.951
	Within Groups	68311697.96	41	1666138.975		
	Total	68318096.47	42			
**contra_Caudate**	Between Groups	135958.148	1	135958.148	0.505	0.481
	Within Groups	11041671.85	41	269309.07		
	Total	11177630	42			
**contra_Putamen**	Between Groups	6569.12	1	6569.12	0.009	0.923

	Within Groups	28742117.16	41	701027.248		
	Total	28748686.28	42			
**contra_Pallidum**	Between Groups	23469.672	1	23469.672	0.273	0.604
	Within Groups	3525264.235	41	85982.055		
	Total	3548733.907	42			
**contra_Amygdala**	Between Groups	604.189	1	604.189	0.007	0.933
	Within Groups	3491076.602	41	85148.21		
	Total	3491680.791	42			
**contra_Accumben_sarea**	Between Groups	780.834	1	780.834	0.089	0.767
	Within Groups	360719.957	41	8798.048		
	Total	361500.791	42			
**contra_VentralDC**	Between Groups	11577.74	1	11577.74	0.042	0.84
	Within Groups	11434536.17	41	278891.126		
	Total	11446113.91	42			
**CC_Posterior**	Between Groups	7809.911	1	7809.911	0.204	0.654
	Within Groups	1572499.159	41	38353.638		
	Total	1580309.07	42			
**CC_Mid_Posterior**	Between Groups	491.503	1	491.503	0.037	0.848
	Within Groups	543617.939	41	13258.974		
	Total	544109.442	42			
**CC_Central**	Between Groups	8.416	1	8.416	0.001	0.977
	Within Groups	405653.026	41	9893.976		
	Total	405661.442	42			
**CC_Mid_Anterior**	Between Groups	1017.855	1	1017.855	0.125	0.726
	Within Groups	333909.82	41	8144.142		

	Total	334927.674	42			
**CC_Anterior**	Between Groups	1451.915	1	1451.915	0.063	0.803
	Within Groups	944596.55	41	23038.94		
	Total	946048.465	42			
**BrainStem**	Between Groups	4212801.528	1	4212801.528	0.424	0.519
	Within Groups	407215527.6	41	9932086.04		
	Total	411428329.2	42			

Key
CC: Corpus callosum
Contra: contralateral
Ipsi: ipsilateral
VentralDC: ventral diencephalon
