# Supplementary material for: Preoperative automated fibre quantification predicts postoperative seizure outcome in temporal lobe epilepsy
Source: Brain. 2016 Nov 15;140(1):68–82. doi: 10.1093/brain/aww280 (PMC5226062; doi:10.1093/brain/aww280)
Supplement: Supplementary Data [file aww280_supp.zip › brain-2016-00271-File010.pdf]

Supplementary Table 2: AFQ results.

Fornix

Param	ROI	Ipsilateral							Contralateral						
		Control	ILAE-1	ILAE-2+	ILAE-1 vs Control		ILAE-2+ vs Control		Control	ILAE-1	ILAE-2+	ILAE-1 vs Control		ILAE-2+ vs Control	
					Cohen's -d	p-value	Cohen's -d	p-value				Cohen's -d	p-value	Cohen's -d	p-value
MD (μm^2 / ms)	1	1.16 (0.30)	1.22 (0.27)	1.43 (0.31)	0.191	0.601	0.885	**0.003**	1.16 (0.30)	1.15 (0.34)	1.20 (0.38)	-0.050	0.906	0.107	0.800
	2	1.39 (0.34)	1.46 (0.29)	1.67 (0.37)	0.215	0.537	0.829	**0.005**	1.39 (0.34)	1.42 (0.44)	1.41 (0.35)	0.109	0.800	0.072	0.886
	3	1.07 (0.25)	1.19 (0.19)	1.32 (0.28)	0.493	0.114	0.967	**0.001**	1.07 (0.25)	1.16 (0.26)	1.14 (0.35)	0.343	0.299	0.246	0.462
	4	1.05 (0.15)	1.22 (0.19)	1.34 (0.26)	1.004	**0.001**	1.584	**<0.001**	1.05 (0.15)	1.06 (0.16)	1.15 (0.28)	0.066	0.891	0.531	0.087
	5	1.14 (0.22)	1.43 (0.18)	1.37 (0.29)	1.369	**<0.001**	0.981	**0.001**	1.14 (0.22)	1.22 (0.22)	1.26 (0.28)	0.387	0.239	0.540	0.082
FA	1	0.22 (0.05)	0.22 (0.04)	0.19 (0.06)	-0.059	0.893	-0.569	0.065	0.22 (0.05)	0.23 (0.05)	0.22 (0.06)	0.283	0.400	0.063	0.891
	2	0.16 (0.04)	0.16 (0.04)	0.15 (0.05)	0.017	0.955	-0.336	0.303	0.16 (0.04)	0.16 (0.04)	0.16 (0.05)	0.021	0.955	-0.016	0.955
	3	0.28 (0.08)	0.28 (0.06)	0.26 (0.09)	-0.042	0.916	-0.189	0.601	0.28 (0.08)	0.27 (0.07)	0.29 (0.09)	-0.062	0.891	0.140	0.730
	4	0.26 (0.06)	0.26 (0.07)	0.24 (0.08)	0.048	0.906	-0.316	0.332	0.26 (0.06)	0.27 (0.05)	0.28 (0.08)	0.253	0.455	0.300	0.364
	5	0.20 (0.05)	0.17 (0.03)	0.18 (0.06)	-0.744	**0.013**	-0.358	0.285	0.20 (0.05)	0.21 (0.04)	0.20 (0.05)	0.120	0.780	0.020	0.955

Parahippocampal white matter bundle

		Ipsilateral							Contralateral						
					ILAE-1 vs Control		ILAE-2+ vs Control					ILAE-1 vs Control		ILAE-2+ vs Control	
Param	ROI	Control	ILAE-1	ILAE-2+	Cohen's -d	p-value	Cohen's -d	p-value	Control	ILAE-1	ILAE-2+	Cohen's -d	p-value	Cohen's -d	p-value
MD (μm^2 / ms)	1	0.98 (0.13)	1.14 (0.21)	1.19 (0.26)	1.024	**<0.001**	1.252	**<0.001**	0.98 (0.13)	0.99 (0.13)	1.08 (0.19)	0.013	0.955	0.656	**0.019**
	2	0.94 (0.10)	1.14 (0.22)	1.18 (0.25)	1.528	**<0.001**	1.663	**<0.001**	0.94 (0.10)	0.95 (0.12)	1.08 (0.19)	0.077	0.854	1.147	**<0.001**
	3	0.99 (0.16)	1.19 (0.29)	1.19 (0.23)	1.039	**<0.001**	1.147	**<0.001**	0.99 (0.16)	0.92 (0.13)	1.11 (0.23)	-0.432	0.137	0.716	**0.010**
	4	1.02 (0.20)	1.24 (0.36)	1.22 (0.23)	0.918	**0.001**	0.963	**0.001**	1.02 (0.20)	0.93 (0.15)	1.07 (0.24)	-0.455	0.114	0.239	0.455
	5	1.07 (0.23)	1.40 (0.40)	1.30 (0.29)	1.199	**<0.001**	0.920	**0.001**	1.07 (0.23)	1.01 (0.19)	1.13 (0.29)	-0.305	0.310	0.246	0.444
FA	1	0.22 (0.06)	0.19 (0.05)	0.21 (0.07)	-0.563	**0.046**	-0.258	0.414	0.22 (0.06)	0.22 (0.05)	0.21 (0.07)	-0.125	0.735	-0.162	0.646
	2	0.26 (0.06)	0.21 (0.06)	0.23 (0.06)	-0.892	**0.001**	-0.494	0.089	0.26 (0.06)	0.26 (0.06)	0.23 (0.07)	-0.026	0.953	-0.552	0.057
	3	0.23 (0.05)	0.20 (0.06)	0.20 (0.05)	-0.516	0.069	-0.396	0.193	0.23 (0.05)	0.25 (0.06)	0.21 (0.06)	0.379	0.205	-0.320	0.299
	4	0.19 (0.05)	0.17 (0.05)	0.17 (0.05)	-0.397	0.183	-0.319	0.299	0.19 (0.05)	0.21 (0.05)	0.20 (0.06)	0.482	0.090	0.304	0.319
	5	0.15 (0.04)	0.12 (0.04)	0.14 (0.05)	-0.675	**0.014**	-0.234	0.459	0.15 (0.04)	0.16 (0.04)	0.16 (0.05)	0.101	0.800	0.089	0.826

Uncinate fasciculus

		Ipsilateral							Contralateral						
					ILAE-1 vs Control		ILAE-2+ vs Control					ILAE-1 vs Control		ILAE-2+ vs Control	
**Param**	**ROI**	**Control**	**ILAE-1**	**ILAE-2+**	**Cohen's -d**	**p-value**	**Cohen's -d**	**p-value**	**Control**	**ILAE-1**	**ILAE-2+**	**Cohen's -d**	**p-value**	**Cohen's -d**	**p-value**
**MD (μm^2 / ms)**	1	0.76 (0.07)	0.86 (0.05)	0.86 (0.07)	1.514	**<0.001**	1.529	**<0.001**	0.76 (0.07)	0.84 (0.06)	0.82 (0.05)	1.239	**<0.001**	0.960	**0.001**
	2	0.73 (0.06)	0.85 (0.09)	0.81 (0.05)	1.818	**<0.001**	1.467	**<0.001**	0.73 (0.06)	0.81 (0.07)	0.80 (0.05)	1.395	**<0.001**	1.338	**<0.001**
	3	0.76 (0.05)	0.90 (0.11)	0.88 (0.08)	2.077	**<0.001**	2.053	**<0.001**	0.76 (0.05)	0.85 (0.05)	0.85 (0.04)	1.845	**<0.001**	1.721	**<0.001**
	4	0.76 (0.07)	0.95 (0.12)	0.93 (0.07)	2.273	**<0.001**	2.495	**<0.001**	0.76 (0.07)	0.86 (0.05)	0.87 (0.07)	1.517	**<0.001**	1.621	**<0.001**
	5	0.77 (0.08)	0.97 (0.11)	0.96 (0.07)	f	**<0.001**	2.405	**<0.001**	0.77 (0.08)	0.87 (0.05)	0.90 (0.09)	1.307	**<0.001**	1.570	**<0.001**
**FA**	1	0.39 (0.06)	0.35 (0.06)	0.35 (0.05)	-0.664	**0.016**	-0.710	**0.011**	0.39 (0.06)	0.37 (0.08)	0.38 (0.04)	-0.343	0.275	-0.154	0.668
	2	0.42 (0.05)	0.36 (0.07)	0.39 (0.05)	-1.120	**<0.001**	-0.594	**0.038**	0.42 (0.05)	0.40 (0.06)	0.39 (0.05)	-0.366	0.239	-0.557	0.059
	3	0.33 (0.04)	0.30 (0.05)	0.31 (0.04)	-0.895	**0.001**	-0.731	**0.009**	0.33 (0.04)	0.33 (0.04)	0.33 (0.04)	-0.094	0.815	-0.052	0.901
	4	0.29 (0.05)	0.26 (0.05)	0.25 (0.04)	-0.527	0.064	-0.786	**0.005**	0.29 (0.05)	0.27 (0.04)	0.28 (0.05)	-0.333	0.290	-0.163	0.648
	5	0.26 (0.04)	0.25 (0.04)	0.23 (0.04)	-0.318	0.299	-0.784	**0.005**	0.26 (0.04)	0.25 (0.05)	0.25 (0.05)	-0.329	0.293	-0.228	0.479
