# Supplementary material for: Preoperative automated fibre quantification predicts postoperative seizure outcome in temporal lobe epilepsy
Source: Brain. 2016 Nov 15;140(1):68–82. doi: 10.1093/brain/aww280 (PMC5226062; doi:10.1093/brain/aww280)
Supplement: Supplementary Data [file aww280_supp.zip › brain-2016-00271-File008.pdf]

Delineation of the fimbria-fornix

Delineation of the fimbria-fornix was performed using in-house scripts written in MATLAB 2012a (Mathworks, Natick, MA) (Glenn et al., 2016), which were based on the procedure followed by AFQ (Yeatman et al., 2012). ROIs were drawn bilaterally along the trajectory of the fimbria-fornix encompassing the dorsal region superior to the anterior thalamus and the ventral region of the anterior mesial temporal lobe. Potential fimbria-fornix fibres were then segmented by identifying all streamlines passing through both inclusion ROIs on a given side. The fimbria-fornix is not included in the probabilistic atlas cross-referenced by AFQ (Hua et al., 2008). Thus to eliminate spurious fibres passing anteriorly between the two inclusion ROIs along the anterior commissure, refinement of the fimbria-fornix was performed using knowledge of its posterior curvature. Cleaning of the fimbria-fornix and computation of tract profiles were created using AFQ's routine (Yeatman et al., 2012). The inclusion ROIs used to identify the fimbria-fornix are overlaid on the International Consortium for Brain Mapping (ICBM) template in Supplementary Figure 1.

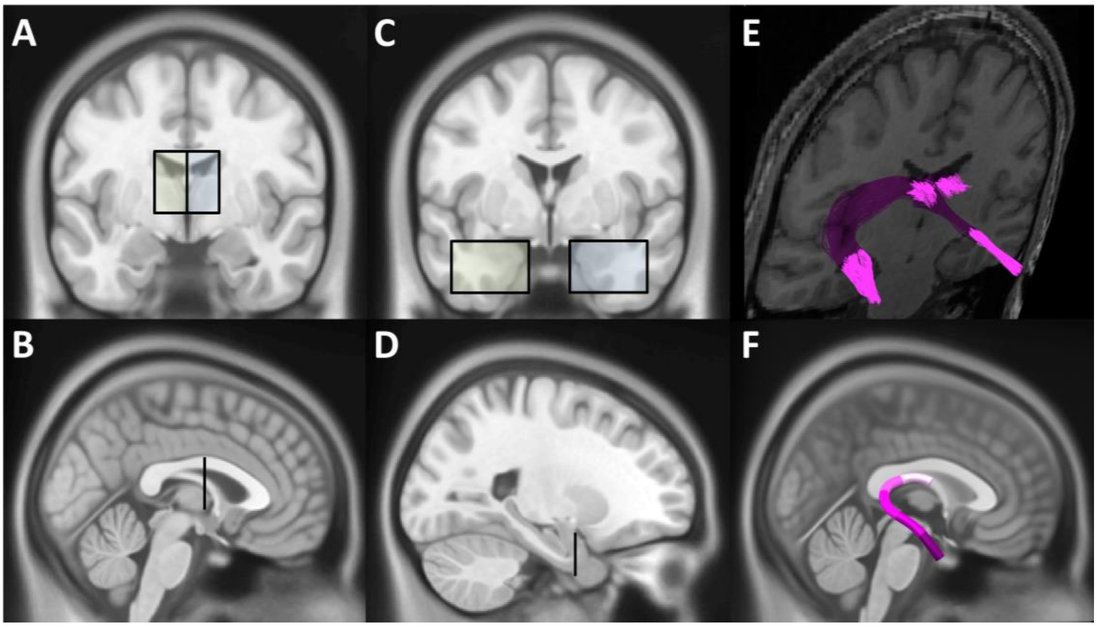

Supplementary Figure 1. (A-D) Bilateral inclusion ROIs for delineation of the fimbria-fornix are demonstrated by the yellow and blue shaded rectangles in the coronal image slices (top row) and the vertical bars in the sagittal image slices (bottom row) encompassing the trajectory of the fimbria fornix from the dorsal region superior to the thalamus (A and B) to the anterior mesial temporal lobe (C and D). (E) Bilateral fimbria-fornix fibres identified for a representative subject. (F) Group-wise representation of the identified fimbria-fornix fibres mapped to the ICBM template for all subjects included in the study, where the five coloured sections represent the five ROIs used for statistical analysis.

References

Glenn GR, Jensen JH, Helpern JA, Spampinato MV, Kuzniecky R, Keller SS, et al. Epilepsy-related cytoarchitectonic abnormalities along white matter pathways. J Neurol Neurosurg Psychiatry. 2016; [Epub ahead of print].

Hua K, Zhang J, Wakana S, Jiang H, Li X, Reich DS, Calabresi PA, Pekar JJ, van Zijl PC, Mori S. Tract probability maps in stereotaxic spaces: analyses of white matter anatomy and tract-specific quantification. Neuroimage. 2008;39:336-47.

Yeatman JD, Dougherty RF, Myall NJ, Wandell BA, Feldman HM. Tract profiles of white matter properties: automating fiber-tract quantification. PLoS One. 2012;7:e49790.
